# Telehealth Behavioral Intervention for Chronic Disease Self-Management in Adults With Physical Disabilities (My Health, My Life, My Way): Protocol for Intervention Fidelity and Dashboard Design for a Randomized Controlled Trial

**DOI:** 10.2196/53410

**Published:** 2024-02-12

**Authors:** Eric Evans, Ayse Zengul, Tejaswini Subhash Chilke, Amy Knight, Amanda Willig, Andrea Cherrington, Tapan Mehta, Mohanraj Thirumalai

**Affiliations:** 1 Department of Health Services Administration School of Health Professions University of Alabama at Birmingham Birmingham, AL United States; 2 Department of Nutrition Sciences School of Health Professions University of Alabama at Birmingham Birmingham, AL United States; 3 UAB Research Collaborative School of Health Professions University of Alabama at Birmingham Birmingham, AL United States; 4 Department of Neuroology Heersink School of Medicine University of Alabama at Birmingham Birmingham, AL United States; 5 Division of Infectious Disease Heersink School of Medicine University of Alabama at Birmingham Birmingham, AL United States; 6 Division of Preventive Medicine Heersink School of Medicine University of Alabama at Birmingham Birmingham, AL United States; 7 Department of Family and Community Medicine Heersink School of Medicine University of Alabama at Birmingham Birmingham, AL United States

**Keywords:** chronic health conditions, telehealth, health coaching, self-management, intervention fidelity protocol

## Abstract

**Background:**

Individuals with physical disabilities experience higher rates of chronic health conditions than individuals without physical disabilities. Self-management programs that use health coaching are effective at eliciting health behavior change in health outcomes such as goal setting, adherence, and health care use. Additionally, web-based resources such as telehealth-based technologies, including SMSS text messaging, web-based applications, and educational multimedia content, can complement health coaching to improve health-related behaviors and the use of health services. The complexity of studies using these resources requires a fidelity protocol to ensure that health behavior studies are administered properly.

**Objective:**

The My Health, My Life, My Way fidelity protocol provides methods, strategies, and procedures of a multifaceted telehealth program for individuals with permanent physical disabilities and chronic health conditions. This health behavior study is a randomized controlled trial with four study arms: (1) scheduled coaching calls with gamified rewards, (2) no scheduled coaching calls with gamified rewards, (3) scheduled coaching calls with fixed rewards, and (4) no scheduled coaching calls with fixed rewards. To guide the fidelity protocol developed, we used the National Institutes of Health Behavior Change Consortium framework (NIH BCC).

**Methods:**

The fidelity intervention protocol was developed by using the 5 primary domains provided by the NIH BCC: study design, provider training, treatment delivery, treatment receipt, and enactment of treatment skills. Following the NIH BCC guidelines and implementing social cognitive theory, this study is designed to ensure that all study arms receive equal treatment across conditions and groups. Health coaches and providers will be trained to deliver consistent health coaching, and thus participants will receive appropriate attention. Educational content will be developed to account for health literacy and comprehension of the material. Multiple fidelity intervention steps such as coaching call logs, regular content review, and participant progress monitoring will translate to participants using the skills learned in their daily lives. Different monitoring steps will be implemented to minimize differences among the 4 treatment groups.

**Results:**

My Health, My Life, My Way has been approved by the institutional review board and will begin enrollment in January 2024 and end in December 2024, with results reported in early 2025.

**Conclusions:**

Intervention fidelity protocols are necessary to ensure that health behavior change studies can be implemented in larger real-world settings. The My Health, My Life, My Way fidelity protocol has used the guidelines by the NIH BCC to administer a telehealth intervention combined with health coaching for individuals with physical disabilities and chronic health conditions. This fidelity protocol can be used as a complementary resource for other researchers who conduct similar research using telehealth technologies and health coaching in real-world settings.

**Trial Registration:**

ClinicalTrials NCT05481593; https://clinicaltrials.gov/study/NCT05481593

**International Registered Report Identifier (IRRID):**

PRR1-10.2196/53410

## Introduction

### Background

According to the US Centers for Disease Control and Prevention, nearly 60% of Americans currently live with a chronic health condition, such as diabetes, heart disease, or hypertension [1]. The prevalence of individual conditions varies and affects activities of daily living differently for each subpopulation. For example, nearly 10% of Americans live with a version of diabetes, including type 1, type 2, and prediabetes [2]. For heart disease, the most common form is coronary artery disease, which is prevalent in the United States in approximately 7% of the population [3]. For example, individuals with physical disabilities experience higher rates of chronic conditions than individuals without physical disabilities [4,5]. Different approaches have been used to manage chronic health conditions and their related symptoms. Prior research has shown that self-management programs, which refer to one’s ability to care for their health, are able to promote positive self-care, increase physical activity, and monitor dietary behavior for individuals with chronic health conditions [6].

Different approaches have been used to provide mechanisms for individuals with chronic health conditions to successfully manage their health. Telehealth interventions have been used as effective strategies for self-management across a variety of chronic health conditions [7-10]. A primary benefit of using telehealth technologies for self-management is the convenience of engaging in programs at flexible times and locations, such as home settings [11,12]. Telehealth programs also provide benefits for clinicians and health care providers, that is, the ability to engage with patients remotely to provide adequate care and promote healthy behaviors. However, there are no accessible and inclusive telehealth programs for individuals with physical disabilities. The lack of inclusive programming for individuals with physical disabilities highlights barriers that this population experiences, including transportation and inadequate time to participate in telehealth interventions [13].

The lack of programming for individuals with physical disabilities demands an immediate remedy. Therefore, we are developing a telehealth program that includes a website with inclusive multimedia educational content combined with access to trained health coaches to provide optimal self-management programming to individuals with physical disabilities. As part of this intervention, we will use an artificial intelligence (AI)–assisted, customized lifestyle program to increase the overall fidelity of the intervention and optimize health coaching resources for telecoaching.

### Reporting Fidelity

To ensure that a health behavior intervention is delivered as intended, it is necessary to develop fidelity protocols to minimize issues with protocol implementation. The complexity of telehealth interventions, the research designs used, and the multiple development teams (health coaches, technical staff, etc) introduce several challenges to maintaining overall study fidelity. Because variations exist in health behavior studies, guidelines have been developed to provide recommendations and strategies for using fidelity concepts in future projects. The National Institutes of Health Behavior Change Consortium (NIH BCC) published recommendations for implementing fidelity concepts for health behavior interventions. The specific areas covered by the NIH BCC include recommendations for study design, provider training, treatment delivery, treatment receipt, and enactment of treatment skills [14]. Therefore, we aim to use the guidelines set forth by the NIH BCC to describe and report the fidelity protocol for the My Health, My Life, My Way study. This intervention will use mobile health technology in conjunction with health coaching to create a comprehensive web-based self-management program for individuals with physical disabilities who experience chronic health conditions. Furthermore, the development of this protocol addresses the 5 domains of fidelity monitoring set forth by the NIH BCC and will provide valuable insight into reproducing future telehealth interventions.

## Methods

### Overview of the Study and Fidelity Protocol

The My Health, My Life, My Way study aims to optimize an AI, telehealth-based self-management program for individuals with physical disabilities and chronic conditions. This program includes a telecoaching website where participants can communicate with health coaches through different communication mediums (ie, texting and phone calls), access customized, educational material related to their specific health conditions, and track health-related behaviors such as physical activity and nutrition. Additionally, the program aims to actively encourage positive lifestyle changes by incorporating features that promote healthy behaviors. These features encompass aiding users in creating well-balanced meal plans and encouraging regular physical activity. By integrating these elements, the program provides a comprehensive toolset for both fostering healthier habits and receiving expert guidance, ultimately contributing to participants’ overall well-being.

Within the website system, AI-embedded technology will be implemented that will have access to an ingredient and recipe database system which will offer personalized dietary suggestions based on the participant’s dietary preferences or restrictions, cooking resources, financial status, and cooking skills. Similarly, the system will be able to provide physical activity recommendations based on health status, preferred activities, and climate. Health coaches will have access to participants’ web-based website to monitor and approve health behavior changes and goals set by the participants. Individuals will be able to communicate with the health coaches through multiple communication mediums, including smartphone apps, SMS text messaging, phone calls, and conversation agents (ie, Amazon Echo). Educational content provided on the website will be created and tailored to individuals with physical disabilities. The combination of these technologies, with a focus placed on the participant, will minimize the time, effort, and costs of using health coaching staff; promote adherence and regular use of the system; and ultimately promote health self-management.

The purpose of this study is to use the Multiphase Optimization Strategy to pilot-test and identify the optimal combination of intervention components that are effective in improving health-related quality of life in adults with physical disabilities and chronic health conditions. We will be using a pilot randomized controlled trial study design, in which 200 adults with physical disabilities and chronic health conditions will be randomized into 1 of 4 groups with 50 participants in each group: (1) scheduled coaching calls with gamified rewards, (2) scheduled coaching calls with independent rewards, (3) no scheduled coaching calls with gamified rewards, and (4) no scheduled coaching calls with independent rewards. Those not receiving scheduled coaching will be able to communicate with health coaches as needed to discuss health-related behaviors and goal setting. Participants will be able to communicate with the health coaches through communication methods such as SMS text messages, phone calls, and the telehealth website. Recruitment will be conducted nationally through web-based advertisements and social media accounts associated with the National Center on Health, Physical Activity and Disability, and ResearchMatch platform. Inclusion criteria include (1) 18 years of age or older; (2) diagnosis of heart disease, chronic lung disease, or type 2 diabetes; (3) living with a permanent physical disability such as spinal cord injury, spina bifida, multiple sclerosis, or stroke; (4) ability to converse and read English; and (5) smartphone or computer access.

Within the intervention, participants will be randomly assigned to 1 of 4 groups (Table 1). The total duration of the program is 24 weeks (6 months). Those receiving scheduling coaching calls will have weekly coaching calls with trained health coaches who will provide customized recommendations and education based on participant needs, including physical activity, nutrition, and other health-related topics. Those not receiving scheduled coaching calls will have the capability to contact health coaches through the telecoaching website, SMS text messages, or phone call to discuss similar health-related objectives. Participants will also receive a physical activity tracker (FitBit) to track activity during the program.

We incorporated the NIH BCC Treatment Fidelity recommendations to develop our fidelity protocol to monitor fidelity and ensure the external and internal validity of the intervention.

**Table 1:** Research design.

Gamification rewards	Coaching calls	
	Scheduled coaching calls	No scheduled coaching calls
Gamification-based rewards	Scheduled coaching calls with gamified rewards	No scheduled coaching calls with gamified rewards
Rewards independent of gamification	Scheduled coaching calls with rewards independent of gamification	No scheduled coaching calls with rewards independent of gamification

### Ethical Considerations

The protocol for the My Health, My Life, My Way study was approved by the University of Alabama at Birmingham's institutional review board (300009485). Enrollment is expected to begin in January 2024 and study completion is expected to end in December 2024.

### Study Design

#### Overview

The principles of the NIH BCC state that fidelity interventions must make certain that (1) appropriate theory and clinical practice are aligned with procedures and implementation, (2) treatment and dose across participants are equal, and (3) possible setbacks in intervention implementation are addressed. Textbox 1 provides the study design and monitoring plan for My Health, My Life, My Way.

**Fidelity of the study design and monitoring plan.**

**Goal**

- Intervention will be congruent with the presented theory and practice
- Ensure equal treatment and dose are provided across and within all conditions
- Implementation setbacks are addressed

**Description from National Institutes of Health Behavior Change Consortium**

- Operationalize treatment to optimally reflect theoretical roots and precisely define variables most relevant to the “active ingredients” of the intervention
- Setbacks in implementation are addressed

**Fidelity plan for My Health, My Life, My Way**

- Monthly review of coaching call checklist
- Monthly review of coaching call logs
- Quarterly assessment of random coaching call audio recordings
- Quarterly assessment of education content provided to participants
- Weekly review of participant journals
- Monthly review of the telecoaching platform and events log
- Quarterly assessment of participant log-ins and time spent on the platform
- Weekly team meetings to discuss participant progress and protocol adherence

#### Presented Theory and Clinical Practices Are Congruent With Procedures and Implementation

My Health, My Life, My Way is grounded in Social Cognitive Theory and is designed to serve as a support system and communication platform among health coaches, participants with disabilities and their caregivers, and health care providers. The website will allow joint efforts for all parties to promote support among friends and family, regular physical activity and healthy dietary behaviors, and goal settings.

#### Equal Dose of Treatments Across Participants

As this study is a 2×2 factorial design, half of the participants will not receive scheduled coaching calls, while the other half will receive scheduled coaching calls. The duration and frequency of coaching calls for the scheduled group will be consistent to ensure an equal dose of calls. Those not receiving scheduled coaching calls will be able to contact the health coaches as needed during the study. For gamification elements, half of the participants will receive incremental rewards for engaging with the study. Such engagement includes but is not limited to (1) engaging with the telehealth website, (2) logging health behavior data, and (3) engaging with educational content. Half will receive independent rewards by performing similar tasks, thereby making the requirements for rewards equal across all participants. Additionally, all groups will be subject to the same data collection methods and study protocols.

#### Protocols for Setbacks in Implementation

All adverse events, emergencies, and issues with participant engagement and intervention will be collected and reported. We will track participant feedback and issues in adherence through the coaching logs and notes collected on the telehealth website, call logs, and staff logs.

### Provider Training

All team members in the study have appropriate backgrounds and experiences in conducting every study activity. The multidisciplinary team includes technical, academic, and programmatic personnel who are able to perform all relevant tasks to ensure the successful completion of the My Health, My Life, My Way study. Academic staff include researchers who are charged with the overall administration of the program and trained health coaches who will engage with participants and provide relevant health-related content such as physical activity and nutrition. Technical staff are trained in developing the website that participants will use and will maintain the technological infrastructure through feedback from academic personnel and participants. Textbox 2 shows the strategies that we will use to monitor provider training based on the NIH BCC.

**Fidelity of provider training.**

**Goal**

- Standardized training
- Ensure provider skill acquisition
- Minimize “drift” in provider skills
- Accommodate provider differences

**Description from National Institutes of Health Behavior Change Consortium**

- Similar training conducted by providers
- Providers trained using predetermined and defined criteria
- Reduce provider skill decay over time
- Ensure adequate training of providers with different levels of skills, experience, and background

**Strategy used in My Health, My Life, My Way**

- Health coaches will receive equal training through specified a curriculum
- Review and monitor coaching calls and call logs
- Evaluate audio recordings of coaching calls
- Collect audio recordings of coaching calls
- Regular team meetings to monitor participant progress and provider protocol adherence

### Treatment Delivery

#### Overview

The NIH BCC provides guidance on how to ensure that interventions are delivered as intended. By using their guidance, we will address four concerns cited by the NIH BCC for treatment delivery: (1) controlling provider differences, (2) reducing differences within study groups, (3) ensuring adherence to study protocols, and (4) reducing contamination between study groups. Textbox 3 shows the strategies used in My Health, My Life, My Way.

**Fidelity strategy for treatment delivery.**

**Goal**

- Control for provider differences
- Reduce differences within study groups
- Ensure adherence to the study protocol
- Reduce contamination between study groups

**Description from National Institutes of Health Behavior Change Consortium**

- Monitor and control for subject perceptions of nonspecific treatment effects (eg, warmth and credibility) across conditions
- Ensure that providers in the same condition are delivering the same intervention
- Ensure that the treatments are being delivered in the way in which they were conceived with regard to content and treatment dose
- Minimize contamination across treatment or control conditions, especially when implemented by the same provider

**Strategies used in My Health, My Life, My Way**

- Coaching call checklist
- Coaching call logs
- Audio recording of coaching calls
- Review of the telecoaching platform and its event log
- Team meetings to discuss participant progress and protocol adherence

#### Controlling Provider Differences

We have developed several strategies to monitor treatment delivery in the study. First, we have developed several coaching guides and scripts that will inform health coaches on what content to cover during coaching calls. We also plan to track the frequency of coaching calls through call logs. This will allow us to evaluate whether participants are receiving an equal number of coaching calls throughout the program. Next, we will collect audio recordings of coaching calls and randomly select calls to evaluate content delivery. The study team will conduct weekly team meetings to evaluate the progress of participants, health coaching curriculum, and protocol adherence.

#### Reducing Differences Within Treatment Groups

This study uses AI to enhance the fidelity of coaching sessions and minimize the effort required by health coaches to provide customized telecoaching. The program will use a telehealth website that will be an assistive tool for health coaches to deliver appropriate content to each participant during coaching call sessions. Health coaches will be provided standardized protocols and guidelines regardless of intervention group assignment. All educational content will be developed by experts to be inclusive of individuals with physical disabilities.

Regardless of group assignment, all procedures will be standardized to reduce differences within treatment groups. Coaches will focus on domains of health, including but not limited to physical activity, mindfulness, and nutrition. In these areas of health, the health coach will follow a progressive routine. For example, if a participant has a goal to increase physical activity participation, the health coach will start with lower activity goals with the ultimate aim of meeting physical activity guidelines of 150 minutes of aerobic activity per week. Similar approaches to nutrition and other health-related behaviors will be used. Although half of the participants will not receive scheduled coaching calls, those participants will be able to communicate with the health coaches through on-demand services through different communication methods regarding health behaviors they seek to improve. Health coaches will follow the same standardized protocols and guidelines for these participants as well.

Finally, the My Health, My Life, My Way telehealth website will include multimedia educational content (ie, videos, infographics, and SMS text messages) to deliver the intervention. All participants will have access to the material and can view them at their discretion.

#### Ensuring Adherence to Treatment Protocols

Health coaches will be given standardized checklists and protocols to ensure adherence to intervention treatment and delivery. The collection of call logs, monthly evaluation of audio recordings, and weekly team meetings will be conducted to further ensure that all protocols are regularly followed. All documents provided to the health coaches will serve as reminders to prepare for any coaching call and discuss relevant content with each participant.

#### Minimizing Contamination Between Conditions

All enrolled participants will be randomized into 1 of 4 groups (Table 1). Each group will have a developed protocol that will be given to the health coaches to use during the study duration. The coaches will use the same educational material that will be adapted for individuals with physical disabilities.

### Receipt of Treatment

A primary element of the success of an intervention is its simplicity and applicability. The NIH BCC emphasizes treatment receipt, which involves assessing the degree to which participants understand the material provided to them and are able to perform health behavior–related skills during the intervention. My Health, My Life, My Way fidelity protocol seeks to achieve goals suggested by the NIH BCC (Textbox 4).

**Fidelity strategy for receipt of treatment.**

**Goal**

- Ensure participant comprehension
- Ensure participant’s ability to use cognitive skills
- Ensure participant’s ability to perform behavioral skills

**Description from National Institutes of Health Behavior Change Consortium**

- Ensure that participants understand the information provided by the intervention
- Make sure that participants are able to use the cognitive skills taught in the intervention
- Make sure that participants are able to use behavioral skills taught in the intervention

**Strategies used in My Health, My Life, My Way**

- Use plain language for content with a target reading at fifth-grade reading level
- Review audio recordings of coaching calls
- Review participant website journals for physical activity, medication, and nutrition behaviors
- Review duration spent on the website by participants
- Conduct study team meetings to review participant progress in the intervention

It is estimated that 80 million US adults have low health literacy, which refers to the inability to listen effectively, read, understand, and interpret text and numerals related to health management, such as food labels, blood glucose measurements, and clinical instructions. Using plain language is important to increase health literacy for individuals with physical disabilities who experience chronic health conditions. As My Health, My Life, My Way is designed for populations with disability, we plan to use language at a fifth-grade reading level for all written content. Such modification to education material is necessary to increase participants’ capacity to perform self-management skills.

My Health, My Life, My Way will incorporate principles of cognitive behavioral therapy to counsel participants on diet, exercise, and positive psychological behaviors like motivation and managing stress. Health coaches will use similar techniques derived from cognitive behavioral therapy to provide proper motivational interviewing that will assist participants in creating and following through on creating goals and implement strategies to meet those goals.

### Enactment of Treatment Skills

The enactment of treatment skills refers to the ability to monitor and increase the capacity of participants to act on behavioral and cognitive skills in real-world settings that they learned during the study intervention [14]. Because we aim to promote long-term adherence to skills beyond the study duration, this program will use adapted and engaging content designed for individuals with physical disabilities in mind. Such content will include multimedia, textual, and graphical material and access through a telehealth website that will provide access to all content (Textbox 5).

**Fidelity strategy for the enactment of treatment skills.**

**Goal**

- Ensure participant’s use of cognitive skills
- Ensure participant’s use of behavioral skills

**Description from National Institutes of Health Behavior Change Consortium**

- Ensure that participants use the cognitive skills provided at the intervention in appropriate life settings
- Ensure that participants actually use the behavioral skills provided in the intervention in appropriate life settings

**Strategies used in My Health, My Life, My Way**

- Review participant logs for physical activity, food, medication, etc
- Review the number of log-ins and time spent on the telehealth website
- Regular team meetings to assess participant progress and overall protocol adherence
- Review and monitor coaching notes collected during the intervention

### My Health, My Life, My Way Telehealth Coaching Dashboard

**Figure 1:**
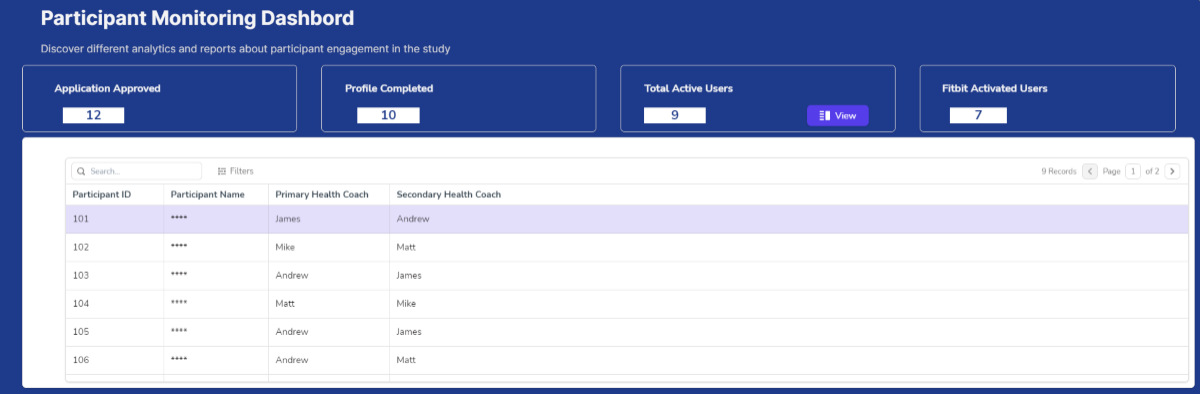
Sample fidelity monitoring dashboard screenshot—participant monitoring dashboard.

**Figure 2:**
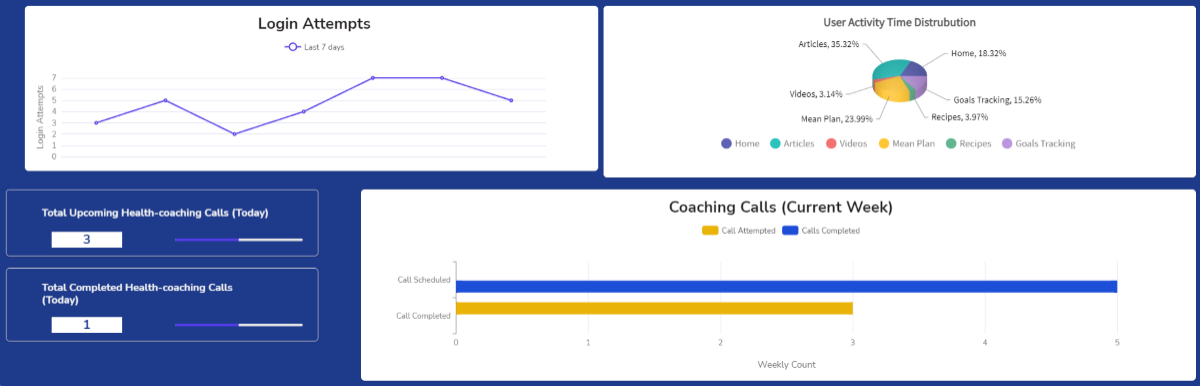
Sample fidelity monitoring dashboard screenshot—log-in analytics.

**Figure 3:**
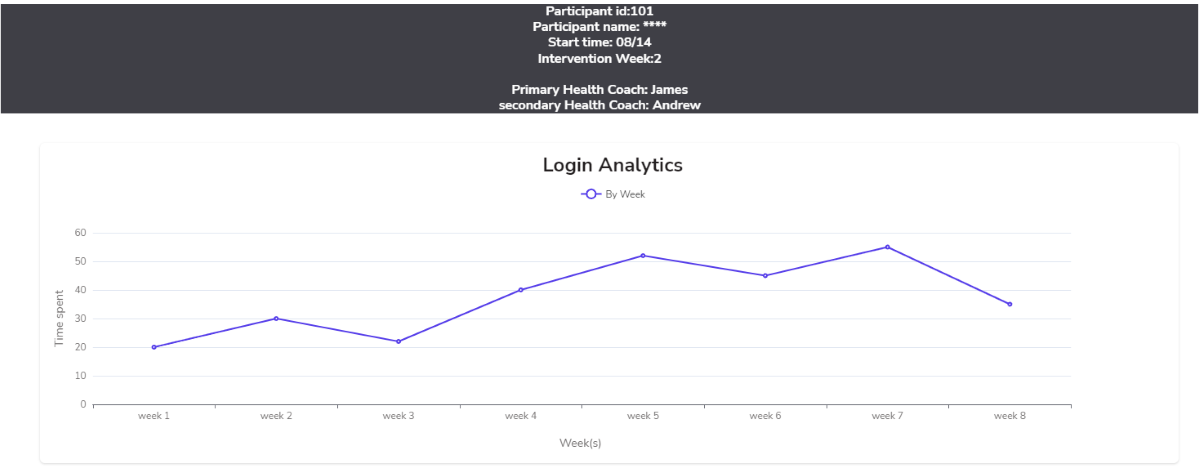
Sample fidelity monitoring dashboard screenshots —summary dashboard.

This program provides a comprehensive telehealth coaching dashboard, a multifaceted solution designed to elevate participant engagement, communication, and monitoring within the realm of health and wellness. This dynamic platform seamlessly integrates 3 essential components. The telehealth coaching dashboard will serve as the pivotal interface enabling participants to establish effective and real-time communication with their designated health coaches. Through a variety of mediums such as SMS text message–based messaging and phone calls, participants can engage in meaningful conversations, seek guidance, and receive personalized support. For participant monitoring and tracking, the platform will offer robust capabilities for monitoring and tracking health-related behaviors, including physical activity, time spent on reading health-related contents, and watching minutes of exercise videos and nutritional choices. This helps health coaches to provide guidance to participants during health coaching sessions. Finally, the user interface for participants will receive an intuitive user interface tailored to their needs. This interface provides them with convenient access to an array of features, such as customized educational materials catering to their specific health conditions, exercise videos, and on-demand health coaching. Moreover, the interface empowers participants to log and manage their activities for promoting positive behavior changes, ranging from effective meal planning to encouraging regular physical activity through goal settings. Overall, through seamless communication channels, comprehensive monitoring tools, and a user-friendly interface, the telehealth coaching dashboard will empower participants to take charge of their health journey while receiving expert guidance and support. Figures 1-3 provide the samples of the telehealth coaching monitoring dashboard.

## Results

Enrollment for the My Health, My Life, My Way program will begin in January 2024 and will end in December 2024. The results of this study will be reported in early 2025.

## Discussion

The purpose of this paper was to outline and describe the intervention fidelity protocol for the My Health, My Life, My Way study. This randomized controlled trial aims to develop and evaluate a telehealth platform, which is paired with inclusive education content for individuals with physical disabilities who experience chronic health conditions. Intervention fidelity refers to mechanisms of monitoring the planned delivery of study interventions [14]. Maintaining strict fidelity throughout study interventions will minimize external influence on both participants and research team members. It will also ensure that results from the intervention are reflective of the participants’ behaviors in real-world settings.

By using strategies provided by the NIH BCC, we developed an intensive fidelity intervention protocol addressing the 5 main domains to monitor, assess, and enhance overall study fidelity. The NIH BCC has provided relevant guidelines and strategies for health behavior interventions to maintain both internal and external validity. Telehealth studies can positively impact self-management skills for individuals with chronic health conditions. The My Health, My Life, My Way program will be the first self-management program for individuals with physical disabilities and chronic health conditions. Because this program is conducted through the web, it has the capability to become accessible, inclusive, scalable, and sustainable. Therefore, by reporting the fidelity intervention protocol for My Health, My Life, My Way, this paper will inform future studies using telehealth technology and health coaching.

The 5 domains to maintain fidelity provided by the NIH BCC addressed in this paper include study design, provider training, treatment delivery, treatment receipt, and enactment of treatment skills. The fidelity monitoring protocol described here was developed in conjunction with a similar telehealth study using similar technology and health coaching for individuals with physical disabilities and diabetes [15,16]. Similar to that fidelity intervention protocol, a challenge of ensuring high fidelity is that My Health, My Life, My Way will use different technological platforms for different fidelity purposes. Examples include using the telehealth website, REDCap (Research Electronic Data Capture; Vanderbilt University) for data collection, and communication platforms for health coaches to engage with participants. Possible implementation setbacks will be addressed, and ongoing collaboration with the multidisciplinary team (ie, research staff, health coaches, and technical support) will reduce variations in fidelity monitoring.

Several steps in program implementation have been conducted prior to fidelity development and standardized protocols have been developed for future steps. In the context of health coaching, strict coaching session guidelines have been developed to reduce variation in conducting health coaching calls, which is a significant concern in telehealth interventions [17,18]. The development of AI that is embedded in our telecoaching website provides convergent communication capabilities that will reduce health coaching workload and need to aid in the personalization of self-management skills for participants. Other monitoring procedures have been created to evaluate the consistency of health coaching sessions, including audio recordings, call logs, and a review of the training used by the coaching team. Such procedures will enhance the internal validity of the protocol and contribute to the creation of effective, evidence-based telecoaching programs.

## Appendix

Multimedia Appendix 1 Peer review report from the National Institute on Disability, Independent Living, and Rehabilitation Research (NIDILRR).

## Abbreviations

AI: artificial intelligence
NIH BCC: National Institutes of Health Behavior Change Consortium framework
REDCap: Research Electronic Data Capture

## References

[1] About chronic diseases. Centers for Disease Control and Prevention.

[2] Zimmet P, Alberti KG, Magliano DJ, Bennett PH (2016). Diabetes mellitus statistics on prevalence and mortality: facts and fallacies. Nat Rev Endocrinol.

[3] Heart disease facts. Centers for Disease Control and Prevention.

[4] (2019). Behavioral risk factor surveillance system—2018 data. Centers for Disease Control and Prevention.

[5] Froehlich-Grobe K, Jones D, Businelle MS, Kendzor DE, Balasubramanian BA (2016). Impact of disability and chronic conditions on health. Disabil Health J.

[6] Odnoletkova I, Goderis G, Nobels F, Fieuws S, Aertgeerts B, Annemans L, Ramaekers D (2016). Optimizing diabetes control in people with type 2 diabetes through nurse-led telecoaching. Diabet Med.

[7] Lee JA, Choi M, Lee SA, Jiang N (2018). Effective behavioral intervention strategies using mobile health applications for chronic disease management: a systematic review. BMC Med Inform Decis Mak.

[8] Whitehead L, Seaton P (2016). The effectiveness of self-management mobile phone and tablet apps in long-term condition management: a systematic review. J Med Internet Res.

[9] Rush KL, Hatt L, Janke R, Burton L, Ferrier M, Tetrault M (2018). The efficacy of telehealth delivered educational approaches for patients with chronic diseases: a systematic review. Patient Educ Couns.

[10] Polisena J, Coyle D, Coyle K, McGill S (2009). Home telehealth for chronic disease management: a systematic review and an analysis of economic evaluations. Int J Technol Assess Health Care.

[11] Bull TP, Dewar AR, Malvey DM, Szalma JL (2016). Considerations for the telehealth systems of tomorrow: an analysis of student perceptions of telehealth technologies. JMIR Med Educ.

[12] Edwards L, Thomas C, Gregory A, Yardley L, O'Cathain A, Montgomery AA, Salisbury C (2014). Are people with chronic diseases interested in using telehealth? a cross-sectional postal survey. J Med Internet Res.

[13] Rimmer JH, Riley B, Wang E, Rauworth A, Jurkowski J (2004). Physical activity participation among persons with disabilities: barriers and facilitators. Am J Prev Med.

[14] Bellg AJ, Borrelli B, Resnick B, Hecht J, Minicucci DS, Ory M, Ogedegbe G, Orwig D, Ernst D, Czajkowski S (2004). Enhancing treatment fidelity in health behavior change studies: best practices and recommendations from the NIH behavior change consortium. Health Psychol.

[15] Zengul A, Evans E, Hall A, Qu H, Willig A, Cherrington A, Thirumalai M (2021). Telehealth behavioral intervention for diabetes management in adults with physical disabilities: intervention fidelity protocol for a randomized controlled trial. JMIR Res Protoc.

[16] Evans E, Zengul A, Hall A, Qu H, Willig A, Cherrington A, Thirumalai M (2021). Disability-inclusive diabetes self-management telehealth program: protocol for a pilot and feasibility study. JMIR Res Protoc.

[17] Willard-Grace R, Chen EH, Hessler D, DeVore D, Prado C, Bodenheimer T, Thom DH (2015). Health coaching by medical assistants to improve control of diabetes, hypertension, and hyperlipidemia in low-income patients: a randomized controlled trial. Ann Fam Med.

[18] Sharma AE, Willard-Grace R, Hessler D, Bodenheimer T, Thom DH (2016). What happens after health coaching? observational study 1 year following a randomized controlled trial. Ann Fam Med.

