# Supplementary material for: Telehealth Behavioral Intervention for Chronic Disease Self-Management in Adults With Physical Disabilities (My Health, My Life, My Way): Protocol for Intervention Fidelity and Dashboard Design for a Randomized Controlled Trial
Source: JMIR Res Protoc. 2024 Feb 12;13:e53410. doi: 10.2196/53410 (PMC10897788; doi:10.2196/53410)
Supplement: Supplementary file 1 [file resprot_v13i1e53410_app1.pdf]

August 5, 2020

Mohanraj Thirumalai, PhD
1716 9th Avenue South
Bitmingham, AL 35294-0111

Applicant Name: ***The University of Alabama at Birmingham***
Application Number: ***DPGE20000200***

Dear Dr. Thirumalai:

Congratulations! Your application to the National Institute on Disability, Independent Living, and Rehabilitation Research (NIDILRR) for consideration under the Fiscal Year 2020 ***Disability and Rehabilitation Research Projects (DRRP): Assistive Technology to Promote Independence and Community Living (DPGE-0415) Grant Competition*** has been selected for funding.

Each application was objectively reviewed, discussed, and scored by a peer review panel. Panel members evaluated applications according to the criteria published in the funding opportunity announcement. I am enclosing a copy of the comment sheets prepared by the peer reviewers. If you wish to discuss the peer review process, the reviewers' comments or any related matters pertaining to your application or NIDILRR's DRRP Program, please contact Stephen Bauer at Stephen.Bauer@acl.hhs.gov or by phone at (202) 795-7304.

Please note that the start date for the grant described in this letter is September 1, 2020.

Thank you for your interest in the DRRP Grants. I look forward to your continued interest in NIDILRR grant programs.

Sincerely,

Kristi Wilson Hill

Kristi W. Hill, PhD
Acting Director
National Institute on Disability, Independent Living, and Rehabilitation Research

Enclosures

Applicant Final Panel Summary Report

Average Score: 95.75

Application Number: DPGE20000200
Application Name: University of Alabama at Birmingham
State: AL City: Birmingham

Criteria Name (Max Score)

1. Importance of the Problem (20 Points)
2. Design of Research Activities (25 Points)
3. Design of Development Activities (25 Points)
4. Design of Dissemination Activities (5 Points)
5. Design of Utilization Activities (5 Points)
6. Plan of Operation (5 Points)
7. Project Staff (10 Points)
8. Adequacy and Accessibility of Resources (5 Points)

TOTAL: 100

Summary of Panel Discussion

Strength:

The overall project was well thought out as reflected in a well-conceptualized logic model, detailing guiding principles, proposed activities, project outputs, and short and long impacts. However, one reviewer noted that this model did not illustrate the role and use of feedback from research participants. The literature review indicates a good understanding of the problems with treatment adherence and motivation of users. Research project R2 will use a multiphase optimization strategy (MOST) to identify and evaluate multiple intervention components (active ingredients) of the telehealth intervention. The proposed 2x2 factorial design for the MOST trial is an excellent choice and appropriate data analysis methods are proposed. The two factor design will evaluate the effects of scheduled coaching calls and use of contingent or non-contingent rewards in gamification. The applicant has committed to permanently deploy the AI4CHRON intervention, at no cost to users, through existing CDC-funded resources of NCHPAD including information specialists/health coaches. This will ensure broad availability and uptake of the intervention. There is a good explanation of how the project furthers the purpose of the Rehabilitation Act by generating new knowledge on community-based self-management interventions that are

designed to optimize health and function of people with disabilities. Detailed outcome measures will examine feasibility of the intervention, user acceptability, intervention fidelity, and intervention efficacy with respect to changes in physical activity, diet, health-related quality of life, self-efficacy, and health outcomes. The proposed analytic techniques will account for multiple comparisons and management of missing data. The principal investigator has an impressive track record of developing and deploying telehealth interventions and strong research and development management experience. The team includes experts in chronic disease management, nutrition, diabetes management, and inclusion specialists. The project has the advantage of exceptional resources available through the partnership of UAB and the Lakeshore Foundation, which is dedicated to the health, wellness, and community integration of people with disabilities. The likelihood of success is also enhanced by the available resources of NCHPAD, which is part of the UAB/Lakeshore Research Collaborative.

Weakness:

It is not clear that the investigators are using feedback from the very large user base of the existing "14 Weeks to a Healthier You" intervention to drive development of the project. It is not clear if the intervention will be accessible and usable by all potential users including those with sensory disabilities. Will disseminated products be available in alternative formats or media? It is not clear how all the "technology" fits together in the AI4CRON. It would improve reader understanding if this process was illustrated using a graphic representation. The use of AI is not clearly explained in the proposal. Specifically, one reviewer expressed concerns that user data privacy had not been adequately addressed. The panel also expressed concerns that the investigators may be underestimating the amount of work that will be required to incorporate AI. The proposal should include a review and discussion (strengths, limitations) of existing commercial solutions (e.g., for diabetes management, physical activity). Some reviewers were not clear on whether the AI4CHRON platform will be developed and tested in a home setting. If not, perhaps a home simulation should be used to ensure the technology fits with the parameters and restrictions (and participants' behavioral patterns) in the home.

General Feedback:

The proposal uses too much italicizing, bold face, and other formatting in an effort to emphasize key information; however, this affected the readability of the application.

Technical Review Form : 1

Criteria Name (Max Score) : Actual Score

1. Importance of the Problem (20 Points) : 20
2. Design of Research Activities (25 Points) : 23
3. Design of Development Activities (25 Points) : 25
4. Design of Dissemination Activities (5 Points) : 5
5. Design of Utilization Activities (5 Points) : 5
6. Plan of Operation (5 Points) : 5
7. Project Staff (10 Points) : 10
8. Adequacy and Accessibility of Resources (5 Points) : 5

TOTAL: 98 / 100

Rationale

Scoring Criteria

Criterion 1: Importance of the Problem

Strength:

Page: 1-8

The applicant provides a detailed description of the target population, people with disabilities who experience chronic health conditions (specifically diabetes, heart and lung disease, and permanent mobility impairments) and the inordinate barriers faced by this population in achieving a healthy lifestyle, including health eating and recommended physical activity.

Page: 13-14

Taking advantage of existing, funded infrastructure and health coach staff of the applicant, the project has the potential for beneficial impact on a broad segment of the target population.

Page: 8-9

The applicant proposes development and efficacy evaluation of "AI4CRHON" an artificial intelligence assisted telehealth intervention for chronic disease management. The project builds on several current or previously-funded (by NIDILRR, PCORI and CDC) research and development efforts, thereby delivering significant return on investment to bring the AI4CRHON platform to market in three years.

Page: 9-12

The overall project is well thought out as reflected in a well-conceptualized logic model, detailing guiding principles, proposed activities, project outputs, and short and long impacts.

Weakness:

None

General:

None

Criterion 2: Design of Research Activities

Strength:

Page: 14-20

Research project R1 will be an ongoing effort to gather input from individuals with disabilities and other stakeholders to shape the proposed research activities. It includes convening a national stakeholder group 12 times over the course of the project and conducting 12 focus groups with different types of participants during the first year. Detailed agendas and group composition are presented for both.

Page: 21-26

Applicant provides a review of literature covering past efforts to apply telehealth interventions to management of chronic health conditions, with an emphasis on well-known challenges of maintaining adherence and engagement. The review also addresses some of the challenges of conducting controlled studies of telehealth interventions, which may include multiple components or "active ingredients" leading to their effectiveness. It is difficult to tease out the importance of each ingredient in the traditional randomized-trial approach.

Page: 23-38

Research project R2 will use a multiphase optimization strategy (MOST) to identify and evaluate multiple intervention components (active ingredients) of the telehealth intervention. The proposed 2x2 factorial design for the MOST trial is an excellent choice and appropriate data analysis methods are proposed. The two factor design will evaluate the effects of scheduled coaching calls and use of contingent or noncontingent rewards in gamification.

Page: 26-27

Appropriate, representative sample of participants will be recruited nationally using the applicant website (with over 45,000 registrants) and social media. from data analysis methods are proposed, including evaluation of intervention effectiveness, costs associated with implementation, and formal cost-effectiveness analysis.

Page: 34-36

Detailed measures employed will examine feasibility of the intervention, user acceptability, intervention fidelity, and intervention efficacy with respect to changes in physical activity, diet, health-related quality of life, self-efficacy, and health outcomes. Proposed analytic techniques appear to be adequate to account for multiple comparisons and management of missing data.

Weakness:

<u>Page: 16; 26</u>

Research questions are limited for R1 and R2. R1 is exploratory so less of a concern. R2 will use the MOST strategy to evaluate different components of intervention. Some hypotheses concerning potential effectiveness of different components could be put forth.

<u>Page: 21-26</u>

Literature review could include a more detailed review of the know successful ingredients of an effective telehealth intervention for self-management (e.g., goal setting, social reinforcement)

General:

Page: 20;37

The appropriate stages of research are identified for each study.

Criterion 3: Design of Development Activities

Strength:

Page: 38-41

A very detailed and thoughtful review of the current state of technology-mediated solutions to chronic disease self-management is presented.

Page: 42-43

The proposed development effort builds on several prior research and development efforts of the team. These efforts include highly successful scaled-up interventions (e.g., 45,000 users of the 14 Weeks to a Healthier You program).

Page: 43-54

A very detailed plan is presented reflecting use of appropriate concepts and components in development of the intervention.

Page: 55-56

Multiple phases of participatory design will be used to ensure appropriate samples are used in usability testing and other development activities.

Page: 56

Development efforts will be performed in appropriate environments under the direction of the PI, who has 18 years of experience building scalable telehealth systems.

Page: 57

Input from individuals with disabilities is assured through companion research efforts (R1) and use of a participatory design approach throughout all development activities.

Weakness:

None

General:

None

Criterion 4: Design of Dissemination Activities

Strength:

Page: 59

The project will benefit from a dissemination plan that takes advantage of the applicants well-established website. The NCHPAD is widely recognized as the preeminent source of health promotion information for people with disabilities and serves over 600k unique visitors annually.

Page: 59

Dissemination efforts are aided by the NCHPAD's extensive presence in social media and supported by the National Association of Chronic Disease Directors

Page: 59-60

The applicant has included a digital marketing budget and proposed continue evaluation and performance improvement of dissemination efforts.

Page: 60-61

The applicant will ensure that dissemination products/activities (website, video and print materials, use of social media) are accessible and understandable to targeted users.

Weakness:

None

General:

None

Criterion 5: Design of Utilization Activities

Strength:

Page: 61

The applicant has committed to permanently deploy the AI4CHRON intervention, at no cost to users, through existing, CDC-funded resources of NCHPAD, including information specialists/health coaches. This will ensure broad availability and potential uptake of the intervention.

Page: 62

The applicant will also engage its network of chronic disease experts and directors in outreach and dissemination efforts to broadcast availability of the no-cost self-management intervention.

Weakness:

None

General:

None

Criterion 6: Plan of Operation

Strength:

Page: 59

Weekly, quarterly, and stakeholder meetings are planned to assess overall progress and gather input from stakeholders. Specific steps are presented for managing project performance to ensure the project remains on track and on budget.

Page: 59-63

Detailed work plans have been established for the project

Page: 63-66

A detailed project plan is presented including key domains of operations and work plans for each project activity. The plan proposes use of an online, shared project management platform (Asana) to track progress across project activities and optimize communications among all project staff.

Weakness:

None

General:

None

Criterion 7: Project Staff

Strength:

Page: 69

The principal investigator has an impressive track record of developing and deploying telehealth interventions and strong research and development management experience.

Page: 70-72

The applicant has included staff with appropriate training and experience in all areas necessary to complete the proposed project activities.

Weakness:

None

General:

None

Criterion 8: Adequacy and Accessibility of Resources

Strength:

Page: 73-75

The project has the advantage of exceptional resources available through the partnership of UAB and the Lakeshore Foundation, which is dedicated to health, wellness, and community integration of people with disabilities.

Page: 75

Likelihood of success is also enhanced by the available resources of NCHPAD, which is part of the UAB/Lakeshore Research Collaborative.

Weakness:

None

General:

None

Technical Review Form : 2

Criteria Name (Max Score) : Actual Score

1. Importance of the Problem (20 Points) : 20
2. Design of Research Activities (25 Points) : 23
3. Design of Development Activities (25 Points) : 25
4. Design of Dissemination Activities (5 Points) : 5
5. Design of Utilization Activities (5 Points) : 5
6. Plan of Operation (5 Points) : 5
7. Project Staff (10 Points) : 10
8. Adequacy and Accessibility of Resources (5 Points) : 5

TOTAL: 98 / 100

Rationale

Scoring Criteria

Criterion 1: Importance of the Problem

Strength:

Page: 32

Very good discussion of how the project fits with the Rehabilitation Act.

Page: 7-8

Excellent presentation of importance of problem. If successful, this project will provide a valuable platform for people with disabilities to improve wellness.

Weakness:

None

General:

None

Criterion 2: Design of Research Activities

Strength:

Page: 65-79

Excellent study design and methodology. Robust sample size. Outlined plans for recruitment and retention. Randomization scheme. Awareness of needs of experimental group in the home (internet, etc). Robust outcome measures and analysis plan.

Weakness:

Page: 54

Sample size may be low and requires stronger justification.

General:

None

Criterion 3: Design of Development Activities

Strength:

Page: 76

Use of advisory group.

Page: 82

Excellent consideration of economics of scaling up project.

Page: 85

Plans to collect robust data about user experiences.

Weakness:

None

General:

None

Criterion 4: Design of Dissemination Activities

Strength:

Page: 58

Well-articulated plan with metrics of success and a variety of dissemination avenues.

Weakness:

None

General:

None

Criterion 5: Design of Utilization Activities

Strength:

Page: 61

Breadth of activities and benchmarks for success are outlined in detail.

Weakness:

None

General:

None

Criterion 6: Plan of Operation

Strength:

Page: 63

The plan of operation is presented in a highly detailed manner, which demonstrates the expertise of the study team. There are specific metrics of success for project goals, which guide the plan of operation.

Weakness:

None

General:

None

Criterion 7: Project Staff

Strength:

Page: 68

Excellent study team with complementary skills and experience.e

Weakness:

None

General:

None

Criterion 8: Adequacy and Accessibility of Resources

Strength:

Page: 73

Excellent, no concerns

Weakness:

None

General:

None

Technical Review Form : 3

Criteria Name (Max Score) : Actual Score

1. Importance of the Problem (20 Points) : 18
2. Design of Research Activities (25 Points) : 24
3. Design of Development Activities (25 Points) : 23
4. Design of Dissemination Activities (5 Points) : 5
5. Design of Utilization Activities (5 Points) : 5
6. Plan of Operation (5 Points) : 5
7. Project Staff (10 Points) : 10
8. Adequacy and Accessibility of Resources (5 Points) : 5

TOTAL: 95 / 100

Rationale

Scoring Criteria

Criterion 1: Importance of the Problem

Strength:

Page: 1-14

The problems are well defined. Clearly stated target population and needs for a systematic approach of sustainable and scalable chronic disease management programs for people with disabilities. Solid previous work on the "14 Weeks to a Healthier You" and CDC's Prevent T2 program. (p6-7) The artificial intelligence assisted telehealth platform is considered highly innovative (p8) Very nice Figure 1 with a logical model for the AI4CHRON development (p10) The proposed project demonstrates a good collaboration between academic and non-profit organization.

Weakness:

Page: 1-14

"This 10-year-old program is perhaps the only available personalized, broad and inclusive option for PWD to engage in a healthier lifestyle until today." (p6) What is the problem of the platform? It would be great to include feedback from current users on the program. Is the AI engine only for diet? How about other self management tasks? (p9) Delivery of the information are all digital technology based. How about people without access to any of those? Will there be a way to deliver by print? This could limit the boarder impact of the proposed technology. (p9)

General:

Page: 1-14

While it is appreciated to highlight keywords and statements by underlining etc. It is a little bit overwhelming with too many highlights.

Criterion 2: Design of Research Activities

Strength:

Page: 14-37

Both R1 (p14-15) and R2 (p20-25) provide a thorough review of current state-of-the-art literature The research questions and aims are clear and theoretical sound. (p16, p26) The sample sizes are justified with appropriate power calculation. (p26) The data analysis plans are detail presented and appropriate. The study protocols and outcome are sufficiently described and justified. People with different disabilities and other stakeholders are included.

Weakness:

Page: 26

It is mentioned that the FitBit will be used to monitor physical activity levels and sleep behaviors, but how the usage of the sleep behaviors are not discussed anywhere. (p28)

General:

None

Criterion 3: Design of Development Activities

Strength:

Page: 38-57

Well thought out development plan with detailed description on key components. User participatory design approach is used with people with disabilities and stakeholders involvement during whole development phases.

Weakness:

Page: 38-57

It is not clear how different technology features of AI4CHRON will be integrated beside Figure 2 (p42). A software diagram or data flowchart could be appreciated. The overlap between the funded NIDILRR support and the proposed work, especially the technology development activities are not described. (p41) In addition, are the features for AI4CHRON driven by another project, or user's needs.

General:

None

Criterion 4: Design of Dissemination Activities

Strength:

Page: 58-61

Good breadth of activities including social media, NCHPAD, NACDD, NARIC and targeted activities towards early adopters. Explicit description of dissemination activities/metrics regarding success shows good planning for this criterion.   Positive history with respect to accessibility to those dissemination channels.

Weakness:

None

General:

None

Criterion 5: Design of Utilization Activities

Strength:

Page: 61-63

The plan is detailed and adequate

Weakness:

None

General:

None

Criterion 6: Plan of Operation

Strength:

Page: 63-68

Plan of operation seems adequate and complete. The tables gave the timeline of activities, and it appears that there are clearly defined roles and responsibilities.

Weakness:

None

General:

None

Criterion 7: Project Staff

Strength:

Page: 68-73

The project staff are highly qualified, experienced, and have a track record of successfully achieving goals of previous grants. The team is diverse both in terms of skills, gender and inclusive of individuals with disabilities. Strong statement of hiring practices for diversity, including Traditionally Underrepresented Groups.

Weakness:

None

General:

None

Criterion 8: Adequacy and Accessibility of Resources

Strength:

Page: 73-75

The team has demonstrated past access and inclusion of individuals with disabilities; and generally has access to clinical populations. The team has extensive past experience in collaborative projects.

Weakness:

None

General:

None

Technical Review Form : 4

Criteria Name (Max Score) : Actual Score

1. Importance of the Problem (20 Points) : 19
2. Design of Research Activities (25 Points) : 20
3. Design of Development Activities (25 Points) : 23
4. Design of Dissemination Activities (5 Points) : 5
5. Design of Utilization Activities (5 Points) : 5
6. Plan of Operation (5 Points) : 5
7. Project Staff (10 Points) : 10
8. Adequacy and Accessibility of Resources (5 Points) : 5

TOTAL: 92 / 100

Rationale

Scoring Criteria

Criterion 1: Importance of the Problem

Strength:

Page: 13

Alignment with Act clearly stated

Page: 8

Need and target group clearly defined

Weakness:

Page: 13

Some of the target population with sensory impairments may not be effectively impacted by the telehealth platform

General:

None

Criterion 2: Design of Research Activities

Strength:

Page: 15

The research described will provide strong evidence and feedback for product development,

Page: 15

Strong review of academic literature

Page: 16

Broad sample base

Page: 20

Analysis is suitable for design

Page: 20

Broad group of participants

Weakness:

Page: 16

Missing research question for R1

Page: 16

It would be useful to know specific details on stakeholder group selection

Page: 19

It was not clear if 10 participants in Phase II focus groups include caregivers or if these were additional

Page: 20,28

R1 or R2 seem closer to development stages 1 & 2. R1 Groups seem to be for customer discovery/product development

General:

Page: 15

Missing from the review are some commercial products offering wellness support

Criterion 3: Design of Development Activities

Strength:

Page: 38

Need and target group well defined and project alignment is clearly shown

Page: 39

Extensive literature review

Page: 45,46

Broad range of options to accommodate many disabilities

Page: 54

Well-articulated focus groups and design feedback

Page: 55, 57

Broad range of participants, likely to give valuable input

Page: 56

Evidence of appropriate environment

Page: 57

Strong design plan and well-articulated stages of development

Weakness:

Page: 40

Apart from PIs previous product, limited number of current products (commercial) reviewed

Page: 54

The use of AI is not clear and the scope of the project may be more significant than expected

General:

Page: 42

Extension of previous work provides strong support for the proposal, however the extent of overlap or duplication was not too clear

Criterion 4: Design of Dissemination Activities

Strength:

Page: 59

Dissemination material are clearly articulated and likely to be effective

Page: 59

Dissemination is appropriate for target group

Page: 59

Addon to existing website, there is clear evidence that dissemination will be high quality and have long term impact

Page: 60

Dissemination will reach broad population

Weakness:

None

General:

None

Criterion 5: Design of Utilization Activities

Strength:

Page: 61

Utilization strategies are clear and well thought out - very likely to be effective

Page: 62

The target group is likely to adopt product

Page: 62

Use of product in other setting was considered

Weakness:

None

General:

None

Criterion 6: Plan of Operation

Strength:

Page: 67

Multiple review/assessment - strong evidence for successful project

Page: 67

Clear well defined plan of operation

Weakness:

None

General:

None

Criterion 7: Project Staff

Strength:

Page: 68

Strong experience and well-suited project team

Page: 68

Evidence of support for underrepresented groups

Page: 69

Team demonstrated broad experience and appropriate training

Weakness:

None

General:

None

Criterion 8: Adequacy and Accessibility of Resources

Strength:

Page: 74

Strong evidence of adequate facilities and resources

Page: 75

Evidence that facilities and resources are accessible

Weakness:

None

General:

None
